# Developing and evaluating machine learning-based risk models for metabolic syndrome among nurses: a cross-sectional study

**DOI:** 10.3389/fpubh.2026.1767549

**Published:** 2026-06-11

**Authors:** Xiaoling Feng, Xinqing Zhu, Jie Peng, Yuanqiu Huang, Huazhen Huang, Yao Tan, Xiaochang Li

**Affiliations:** Department of Nursing, The Sixth Affiliated Hospital of Guangxi Medical University, Yulin, China

**Keywords:** machine learning, metabolic syndrome, nurses, occupational health, prediction model

## Abstract

**Background:**

Metabolic syndrome is a group of risk factors that increase the risk of cardiovascular disease. Nurses have a higher risk of metabolic syndrome due to high work pressure and irregular lifestyle. Early recognition of metabolic syndrome is essential to prevent related complications and improve nurse health.

**Methods:**

A cross-sectional survey was used to collect valid questionnaire data of 900 nurses. Univariate analysis and multivariate Logistic regression analysis were used to screen the key variables. Logistic regression, random forest, XGBoost, support vector machine and neural network were used to predict and evaluate the performance of the model. SHAP values were also used to explain the importance of model features. In addition, 227 nurses were collected for external validation of the optimal model.

**Results:**

The random forest model had the best performance on the test set [AUC = 0.890 (0.855–0.925)], and the AUC of the external validation was 0.80 [0.762–0.838]. Among them, BMI, job burnout, shift work, age and other 9 characteristics were the key factors affecting the prediction.

**Discussion:**

The superior performance of the random forest model and identification of BMI and job burnout as core predictors provide evidence-based support for integrating machine learning-based screening into nurse occupational health policies, enabling early identification and targeted intervention for high-risk nurses.

**Conclusion:**

This study successfully established the prediction model of metabolic syndrome in nurses. The random forest model has high accuracy. The key factors identified provide theoretical support for early intervention, provide quantitative tools for identifying high-risk nurses, support personalized health intervention, and help to improve the overall health level and work efficiency of nurses.

## 1 Introduction

Metabolic syndrome (MetS) is a group of metabolic disorders characterized by central obesity, hypertension, insulin resistance, and dyslipidemia, which significantly increases the risk of cardiovascular disease and type 2 diabetes (1, 2). With lifestyle changes, the global prevalence of adult MS has reached 20%−40%, and continues to rise, becoming a major public health challenge (3–5).

Among medical practitioners, nurses are at high risk of metabolic syndrome due to their occupational characteristics such as long-term shift work, high-intensity work load, work and rest disorder, and lack of physical activity (6). Epidemiological studies have shown that the proportion of nurses suffering from metabolic syndrome is significantly higher than that of the general population, and the risk of disease will gradually increase with the increase of service years (7–9).

Metabolic syndrome will not only have a negative impact on the physical and mental health of nurses, but also reduce the quality of care, and may even lead to job burnout and turnover intention (7, 10, 11). Therefore, early identification and intervention of metabolic syndrome has become an important topic in nursing management. The development of an effective prediction model can help nursing managers identify high-risk nurses as early as possible, implement corresponding health management and intervention, so as to reduce the occurrence of metabolic syndrome and improve the quality of nursing work and the job satisfaction of nurses.

In recent years, the rapid development of machine learning technology has provided a new paradigm for risk prediction of metabolic syndrome. Compared with traditional statistical models, machine learning algorithms can identify potential predictors through feature importance ranking, and use non-linear modeling ability to mine the interaction effect between variables (12, 13).

Although several studies have explored factors affecting metabolic syndrome, such as work stress, lifestyle, and genetic factors, there is a lack of systematic and accurate predictive models (8, 14). This study aims to develop and validate a predictive model for metabolic syndrome in nurses using a variety of statistical and machine learning methods. This study aimed to identify key predictors to better understand which variables significantly contribute to the development of metabolic syndrome. Through this model-based analysis, not only can the accuracy of prediction be improved, but it can also provide a scientific basis for care managers and healthcare providers to develop more targeted intervention strategies.

The main objectives of this study were as follows: (1) to identify the key factors affecting metabolic syndrome in nurses by univariate analysis and multivariate Logistic regression analysis; (2) apply and compare various machine learning models, such as Logistic regression, random forest, XGBoost, support vector machine, and neural networks, to predict, validate, and identify the best prediction model for metabolic syndrome in nurses to provide valuable insights for future nursing management; Ultimately, this study aims to promote attention to and intervention for nurses' overall health and work efficiency.

## 2 Methods

### 2.1 Study design

In this study, a cross-sectional survey was conducted to collect data from nurses in six tertiary hospitals by questionnaire. Based on these data, a predictive model for metabolic syndrome was constructed. In addition, according to the same inclusion and exclusion criteria, convenience sampling method was used to extract a part of the nurses' data from two other tertiary hospitals in China as the data set for external validation. Ethical guidelines were followed in this study, and all participants participated voluntarily and gave informed consent.

### 2.2 Study participants

The subjects of the study were nurses from tertiary hospitals in China. The inclusion criteria were as follows: (1) age > 18 years old; (2) having a nurse's professional qualification certificate; (3) voluntarily participated in this study and signed informed consent. Exclusion criteria: (1) nurses who were on study leave, vacation, maternity leave, or breastfeeding during the investigation period; (2) MS secondary to other diseases; (3) complicated with major diseases or cancer. All participants signed an informed consent form before participating in the study. In the model construction phase, 900 valid questionnaires were collected, and in the model validation phase, 227 valid questionnaires were collected, and 1,127 valid questionnaires were collected.

Sample size determination was guided by the principle of maintaining sufficient statistical power for both multivariate logistic regression and machine learning model development. With 30 independent variables under consideration and aiming for 5–10 events per variable (EPV) to ensure stable estimation, we targeted a minimum of 750 participants for the modeling phase. Considering the need for external validation (targeting 200+ participants) and anticipating approximately 25% potential missing data based on our pilot study, we aimed to recruit 1,200 nurses. Ultimately, we obtained 1,127 valid questionnaires (93.9% response rate), with 900 used for model development and 227 for external validation, which exceeded our minimum requirement and ensured robust analysis.

A total of 931 nurses from six tertiary hospitals were initially enrolled in 2024. After excluding 31 participants (2.7%) with incomplete key variables, 900 nurses were included in the final analysis. The dataset (*n* = 900) was split into training (70%, *n* = 630) and testing (30%, *n* = 270) sets. An additional 227 nurses from two independent hospitals were used for external validation.

### 2.3 Data collection

All metabolic parameters were obtained from routine occupational health examinations conducted within 3 months of questionnaire administration, extracted from hospital electronic medical records. Nurses were classified as having metabolic syndrome if they met ≥3 criteria, or not having metabolic syndrome.

Data were collected through self-administered questionnaires covering demographic variables (e.g., age, sex, and education), sleep quality, work-related variables (e.g., years worked, number of night shifts per week, and department worked), and psychological variables (e.g., job burnout and job stress). Psychological variables included job burnout. MBI job burnout scale was used, including 3 dimensions, 12 items and 7-point Likert scale (15). Occupational stress was evaluated by a job stress scale containing 2 dimensions and 11 items, and a 5-point Likert scale (16). The Pittsburgh Sleep Quality Index (PSQI) was used for sleep quality, which consisted of 19 self-rated items and 5 patient-rated items (17). In this study, the PSQI scale showed good reliability, and its Cronbach's α coefficient reached 0.852. The Cronbach's α coefficients of the three dimensions of the Maslach Burnout Inventory were 0.931, 0.910 and 0.923, respectively, indicating that the measurement tool had a high internal consistency. The Cronbach's α coefficient of the time stress dimension of the occupational stress scale was 0.899, and the Cronbach's α coefficient of the anxiety dimension was 0.821. All these scales demonstrated high reliability, ensuring the validity and reliability of the data collected.

To minimize potential biases, we employed several strategies: selection bias was reduced by recruiting participants from multiple tertiary hospitals across different regions; information bias was addressed through the use of validated, standardized questionnaires and anonymous data collection to encourage honest responses; and measurement bias was controlled by training research assistants in standardized data collection procedures and conducting a pilot study with 50 nurses to ensure clarity of questionnaire items prior to formal data collection.

### 2.4 Definitions of independent and dependent variables

Dependent variable: the dependent variable in this study was whether the nurse had metabolic syndrome or not. Metabolic syndrome was defined according to the Chinese Diabetes Society (CDS) 2004 diagnostic criteria, requiring the presence of three or more of the following five components: the Chinese Diabetes Society (CDS) recommended diagnostic criteria of metabolic syndrome: Central obesity: waist circumference ≥90 cm for men, ≥85 cm for women; Hyperglycemia: fasting blood glucose ≥6.1 mmol/L or 2 h postprandial blood glucose ≥7.8 mmol/L and/or diagnosed as diabetes mellitus and treated. Hypertension: blood pressure ≥130/85 MMHG (1 mmHg = 0.133 kPa) and/or hypertension was confirmed and treated. fasting triglyceride (TG) ≥ 1.70 mmol/L; Fasting HDL-C < 1.04 mmol/L. The nurses were divided into two groups: those with and those without metabolic syndrome. Independent variables: Thirty independent variables were analyzed in this study, including: age, gender, education, marital status, number of children, weekly exercise frequency, smoking status, passive smoking status, frequency of tea/coffee intake, frequency of alcohol intake, frequency of dairy product intake, frequency of dessert intake, eating habits, eating preferences, frequency of late-night snacks, daily water intake, working years, department, professional title, scientific research/teaching position Job, monthly income, whether to shift at night, the number of night shifts per month, BMI, whether to have family history of cardiovascular disease, self-conscious health status, PSQI total score, whether to have the habit of nap, occupational stress, job burnout, etc.

#### 2.4.1 Operationalization of key predictors

BMI: calculated as weight (kg)/height (m)^2^ based on measurements from occupational health examinations, entered as continuous variable.

Age: collected in years from identity documents, entered as continuous variable.

Shift work: participants were categorized into the following types of shift pattern: no shift work (i.e., standard office hours) and rotating (including night).

### 2.5 Statistical methods

Univariate analysis and multivariate Logistic regression analysis were used to identify the key variables affecting metabolic syndrome. Statistical significance was set at a *P*-value < 0.05. Missing data were minimal, with < 3% missing for any single variable. Given the low proportion of missingness, we employed listwise deletion for the primary analyses, which resulted in exclusion of 31 participants (2.7%) with incomplete key variables. Sensitivity analyses using multiple imputation (*m* = 20) confirmed that results were robust to the missing data approach. Multiple machine learning models, such as Logistic regression, random forest, XGBoost, support vector machine and neural network were used to verify the effectiveness of the prediction model. The dataset was divided into a training set and a test set at a ratio of 7:3, and 10 repeated samples were performed to reduce model error. The model evaluation indexes included accuracy, precision, recall, F1 score, AUC and specificity. To further account for the features in the model, shap (Shapley Additive ExPlanations) values were used to analyze the contribution of each variable to the predicted outcome. SHAP value plots were generated to illustrate the importance of different variables and their positive or negative effects on metabolic syndrome.

### 2.6 Statement of ethics

The study was approved by the hospital ethics committee. This study strictly adhered to the ethical requirements of the Declaration of Helsinki, all participants signed an informed consent form, and the study process strictly adhered to ethical guidelines to ensure the privacy and data security of the participants.

## 3 Results

A total of 900 nurses from six tertiary hospitals in China were investigated. The sample included 900 participants, mainly female (94.78%), with an average age of 30.78 (±6.81) years, and most of them were undergraduates, accounting for 70%.

The key independent variables affecting metabolic syndrome were identified by univariate analysis and multivariate regression analysis. The results of univariate analysis showed that 17 independent variables could affect the occurrence of metabolic syndrome in nurses. The data included age, education level, number of children, weekly exercise frequency, exposure to second-hand smoke, frequency of tea/coffee intake, frequency of alcohol intake, frequency of dessert intake, dietary habits, dietary preferences, daily water intake, working years, professional title, shift work, BMI, family history of cardiovascular disease, and job burnout. See Table 1.

**Table 1:** Relationship between general information of nurses and metabolic syndrome.

Variable	Item	MS Group (*n* = 172)	Non-MS group *(n* = 728)	*X^2^*/*t*	*P*
Age/years		33.86 ± 8.017	30.06 ± 6.289	−5.768	< 0.001
Gender	Male	13	34	2.344	0.126
	Female	159	694		
Education	College or below	41	224	14.709	< 0.001
	Undergraduate	127	503		
	Master's degree or above	1	4		
Marital status	Unmarried	65	329	3.614	0.164
	Married	104	392		
	Divorced/widowed	3	7		
Number of children	0	82	389	8.392	0.039
	1 of them	40	138		
	Two of them	44	194		
	>Two of them	7	6		
Number of exercises per week	0	107	433	6.164	0.046
	1–3 times	59	228		
	>3 times	6	67		
Smoking	Yes	3	11	0.049	0.824
	No	169	717		
Effects of second-hand smoke	Yes	106	510	4.575	0.032
	No	66	218		
Tea/Coffee	Hardly/never	73	395	8.383	0.015
	Occasionally	24	93		
	Often	75	240		
Wine	Hardly/never	142	645	10.470	0.005
	Occasionally	23	45		
	Often	7	38		
Dairy products	Hardly/never	29	116	0.473	0.789
	Occasionally	34	161		
	Often	109	451		
Dessert	Hardly/never	18	116	10.930	0.004
	Occasionally	33	200		
	Often	121	412		
Eating habits	Regular meals	92	457	5.043	0.025
	Eating disorders	80	271		
Food preferences	No adverse dietary preferences	81	459	14.760	< 0.001
	Oily/salty/sweet	91	269		
Late night snack	Don't eat	13	70	1.482	0.477
	Eat once in a while	114	494		
	Eat often	45	164		
Daily water intake/mL	< 500	10	11	18.961	0.002
	500–999	48	173		
	1,000–1,499	51	298		
	1,500–1,999	51	179		
	2,000–2,499	11	58		
	≥2,500	1	9		
Working years/year		11.76 ± 8.291	8.42 ± 6.465	−4.941	< 0.001
Department of Medicine	Internal medicine	71	280	10.770	0.215
	Surgery	34	173		
	Intensive care unit	18	86		
	Emergency Department	4	37		
	Pediatrics	12	39		
	Obstetrics and gynecology	10	33		
	Operating room	11	20		
	Outpatient service	1	8		
	Other	11	52		
Professional title	Nurse	31	184	48.354	< 0.001
	Nurse practitioner	50	311		
	Nurse in charge	62	204		
	Associate chief nurse or above	29	29		
Research/teaching tasks	Yes	73	266	2.065	0.151
	No	99	462		
Monthly income/yuan	< 4,000	19	102	1.748	0.626
	4,001–6,000	80	304		
	6,001–9,000	54	238		
	>9,000	19	84		
Shift work	Yes	145	560	4.464	0.035
	No	27	168		
Number of night shifts per month	0	43	152	1.987	0.575
	≤ 3	29	112		
	4–5	72	332		
	≥6	28	132		
BMI		23.91 ± 4.142	21.14 ± 2.964	−8.274	< 0.001
Family history of cardiovascular disease	Yes	84	424	5.005	0.025
	No	88	304		
Perceived health status	Very good	17	111	3.297	0.348
	Better off	55	222		
	In general	83	327		
	Worse off	17	68		
PSQI		8.42 ± 4.075	8.80 ± 3.807	−1.150	0.251
Nap in the afternoon	Yes	73	313	0.017	0.895
	No	99	415		
Occupational stress		20.62 ± 5.651	18.55 ± 6.536	−4.196	0.320
Burnout at work		84.44 ± 9.395	79.14 ± 8.444	−6.770	0.013

Multivariate Logistic regression analysis was performed on 17 significant independent variables in univariate analysis to further control the confounding factors, and finally determined that age, weekly exercise frequency, alcohol intake frequency, dietary preference, daily water intake, professional title, work shift, BMI, and job burnout were significant factors (*P* < 0.05). It is suggested that these factors play an important predictive role in metabolic syndrome. See Table 2.

**Table 2:** Multivariate Logistic regression analysis of metabolic syndrome in nurses.

Variable	β	SE	Wald	*P*	OR	95% CI
Age/years	0.161	0.046	12.306	< 0.001	1.174	(1.073–1.284)
Education			
Undergraduate	−1.905	1.411	1.821	0.397	0.149	(0.009–2.367)
Master's degree or above	−1.832	1.409	1.691	0.193	0.160	(0.010–2.533)
Number of children			
1 of them	−0.618	0.824	0.563	0.058	0.539	(0.107–2.711)
Two of them	−0.978	0.821	1.417	0.234	0.376	(0.075–1.881)
≥Two of them	−1.368	0.808	2.868	0.090	0.255	(0.052–1.240)
Number of exercises per week			
1–3 times	−1.310	0.495	7.015	0.030	3.705	(1.406–9.766)
>3 times	−1.155	0.501	5.311	0.021	3.175	(1.189–8.481)
Tea/Coffee			
Occasionally	−0.450	0.239	3.555	0.145	0.637	(0.399–1.018)
Often	−0.072	0.328	0.048	0.826	0.931	(0.489–1.770)
Wine			
Occasionally	0.953	0.556	2.940	0.047	2.593	(0.873–7.706)
Often	1.521	0.628	5.870	0.015	4.575	(1.337–15.653)
Dessert			
Occasionally	−0.372	0.320	1.354	0.080	0.689	(0.368–1.290)
Often	−0.575	0.270	4.552	0.245	0.562	(0.331–0.954)
Eating habits	0.037	0.219	0.029	0.865	1.038	(0.676–1.594)
Food preferences	0.529	0.216	6.010	0.014	0.589	(0.386–0.899)
Daily water intake/ml			
500–999	−1.388	1.241	1.252	0.034	0.107	(0.352–45.581)
1,000–1,499	0.210	1.138	0.853	0.042	1.234	(0.133–11.488)
1,500–1,999	−0.223	1.134	0.844	0.039	0.800	(0.087–7.377)
2,000–2,499	0.385	1.136	0.735	0.115	1.469	(0.159–13.607)
≥2,500	−0.300	1.186	0.800	0.064	0.741	(0.073–7.568)
Working years/year	−0.082	0.049	2.783	0.095	0.921	(0.836–1.015)
Professional title			
Nurse practitioner	1.094	0.685	2.555	0.044	0.335	(0.087–1.281)
Nurse in charge	1.447	0.576	6.305	0.012	0.235	(0.076–0.728)
Associate chief nurse or above	0.959	0.486	3.890	0.049	0.383	(0.148–0.994)
Shift work	0.799	0.290	7.591	0.006	0.450	(0.255–0.794)
BMI	0.205	0.034	36.066	< 0.001	1.228	(1.148–1.313)
Family history of cardiovascular disease	−0.114	0.214	0.286	0.593	0.892	(0.586–1.357)
Burnout at work	0.057	0.012	23.652	< 0.001	1.058	(1.034–1.083)

**Figure 1:**
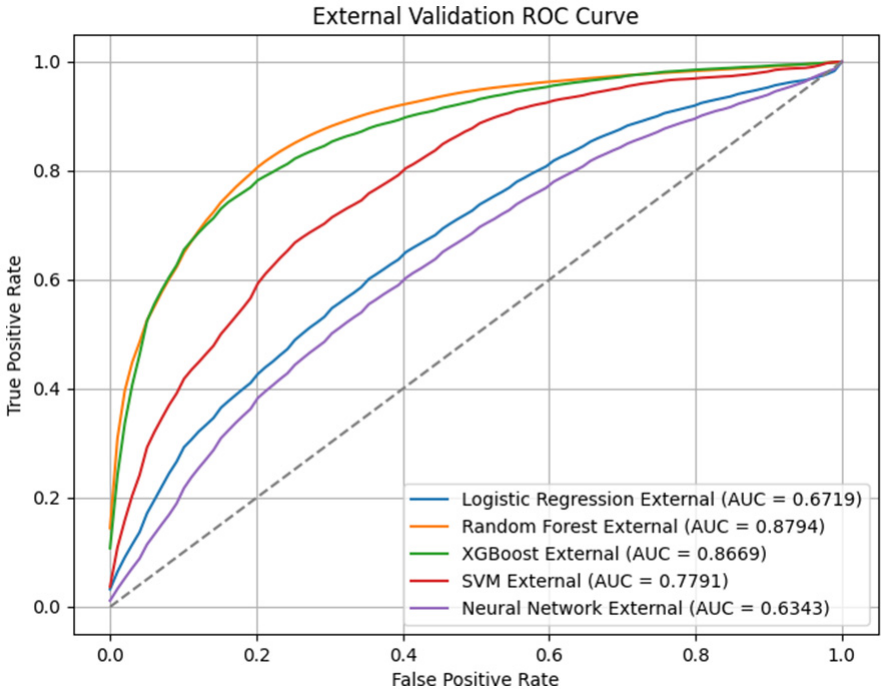
ROC curves on the test set of five models for machine learning.

In this study, we trained five models, including Logistic regression, random forest, XGBoost, support vector machine, and neural networks, and the data were divided into training and test sets at a ratio of 7:3, followed by 10 repeated sampling. The model was evaluated by area under the curve (AUC), and the results showed that random forest and XGBoost had high AUC values and performed well on the training set. See Table 3. On the internal validation set, the random forest model had the highest prediction accuracy, precision and AUC value, which were 0.869, 0.763 and 0.879, respectively. The XGBoost model had the highest F1 score of 0.593, the support vector machine model had the highest recall rate of 0.680, and the neural network had the highest specificity of 0.988. The random forest model performs very well on multiple metrics, especially on the test set. See Table 4. The ROC curve on the internal validation set of this study is shown in Figure 1. Therefore, the random forest model can be considered as the best model in this analysis.

**Table 3:** Performance comparison of five machine learning algorithms in predicting ms risk of nurses on training sets.

Model	Accuracy	Precision	Recall	F1	AUC	Specificity
Mean (95% CI)	
Logistic Regression	0.592 (0.571–0.613)	0.272 (0.222–0.322)	0.696 (0.581–0.810)	0.390 (0.325–0.454)	0.680 (0.636–0.725)	0.566 (0.544–0.588)
Random Forest	0.843 (0.820–0.866)	0.659 (0.587–0.732)	0.388 (0.324–0.453)	0.481 (0.438–0.524)	0.869 (0.855–0.884)	0.951 (0.934–0.969)
XGBoost	0.840 (0.821–0.858)	0.615 (0.537–0.693)	0.445 (0.424–0.466)	0.512 (0.489–0.536)	0.845 (0.817–0.874)	0.934 (0.916–0.951)
SVM	0.729 (0.713–0.745)	0.373 (0.316–0.430)	0.670 (0.542–0.797)	0.476 (0.404–0.549)	0.783 (0.732–0.833)	0.741 (0.711–0.771)
Neural Network	0.806 (0.776–0.836)	0.413 (0.074–0.753)	0.044 (0.009–0.079)	0.077 (0.018–0.136)	0.638 (0.617–0.658)	0.986 (0.978–0.995)

**Table 4:** Performance comparison of five machine learning algorithms in predicting MS risk of nurses on the test set.

Model	Accuracy	Precision	Recall	F1	AUC	Specificity
Mean (95% CI)	
Logistic regression	0.589 (0.588–0.591)	0.272 (0.270–0.273)	0.672 (0.668–0.676)	0.386 (0.385–0.388)	0.672 (0.669–0.674)	0.570 (0.568–0.572)
Random forest	0.869 (0.868–0.870)	0.763 (0.759–0.768)	0.470 (0.465–0.474)	0.578 (0.574–0.582)	0.879 (0.878–0.881)	0.964 (0.963–0.965)
XGBoost	0.868 (0.867–0.869)	0.731 (0.727–0.736)	0.504 (0.500–0.509)	0.593 (0.590–0.597)	0.869 (0.868–0.871)	0.955 (0.954–0.956)
SVM	0.725 (0.723–0.727)	0.382 (0.380–0.384)	0.680 (0.676–0.684)	0.488 (0.486–0.490)	0.779 (0.778–0.781)	0.736 (0.734–0.738)
Neural network	0.804 (0.803–0.804)	0.301 (0.282–0.320)	0.031 (0.029–0.034)	0.053 (0.049–0.057)	0.632 (0.630–0.635)	0.988 (0.987–0.989)

**Figure 2:**
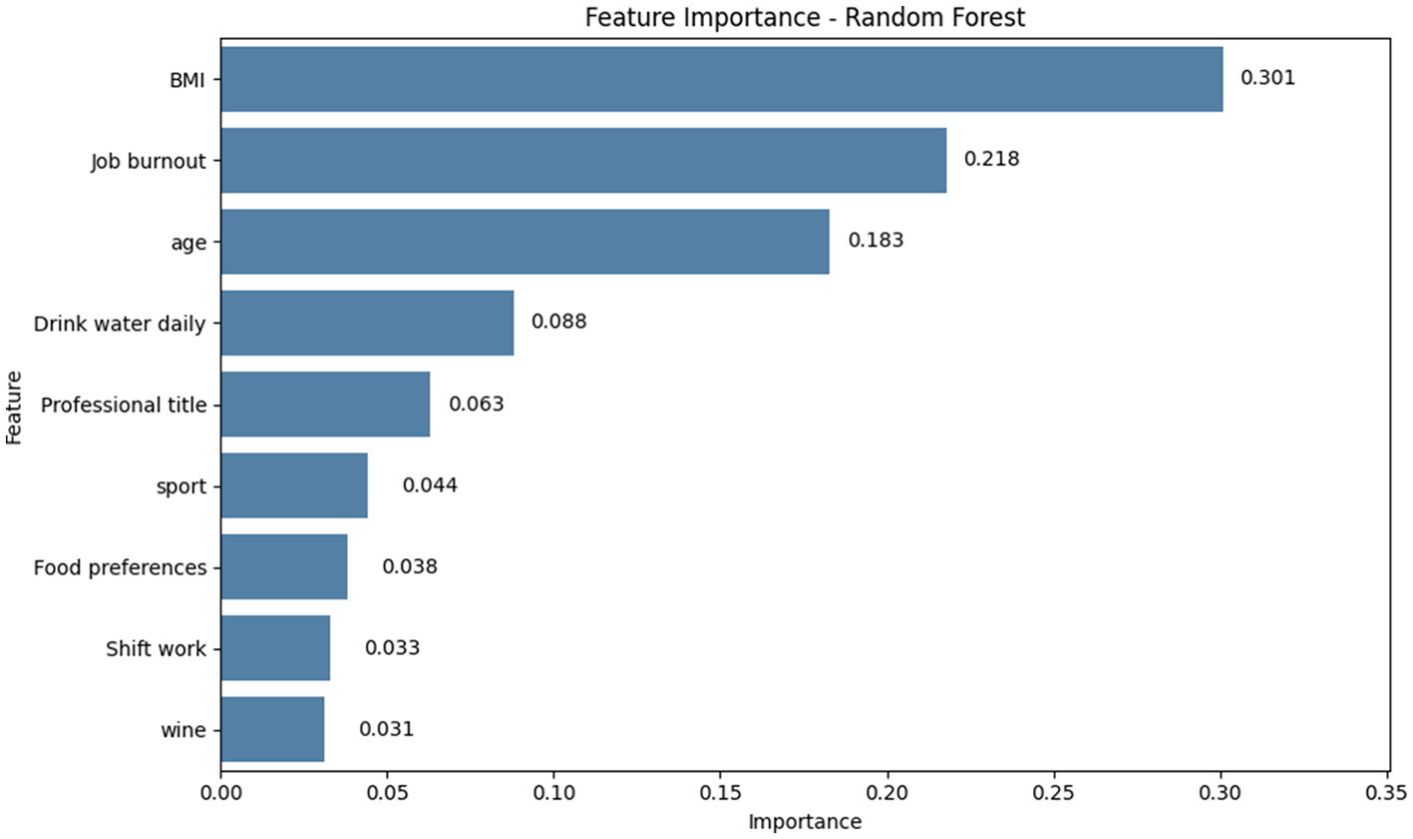
Feature importance of the random forest model.

At the same time, we output the importance of each feature in the data set in the optimal model (random forest) in model classification, that is, the degree of influence of different features on metabolic syndrome of nurses. The feature importance of model prediction results is output and ranked to be more intuitive, and the feature importance map of random forest model is drawn, as shown in Figure 2.

**Figure 3:**
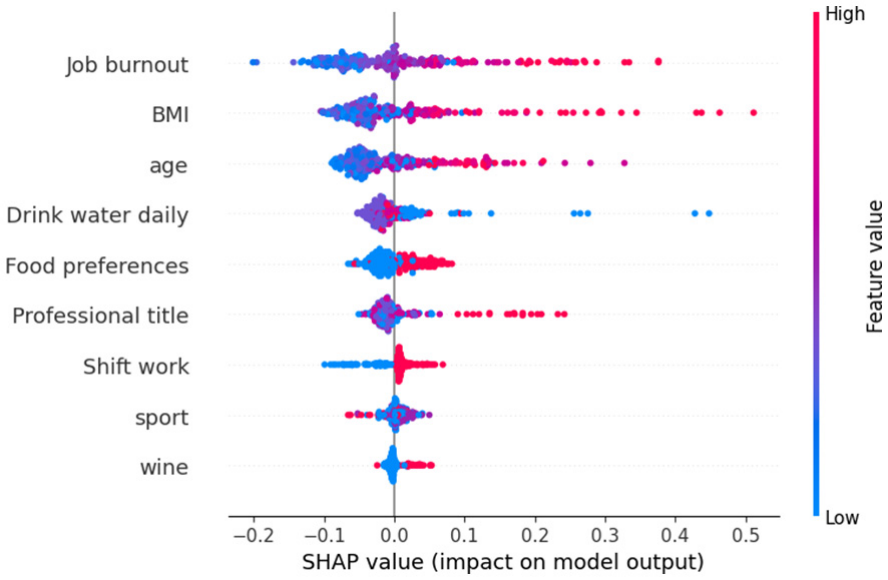
Density scatter plot of the global interpretation of the random forest model SHAP.

The density scatter plot of SHAP global interpretation, the horizontal axis is SHAP value, each row represents a feature, blue and red indicate low and high feature values, respectively. In the random forest model, for example, higher Job burnout values will push up the predicted value of the risk of metabolic syndrome, see Figure 3.

**Figure 4:**
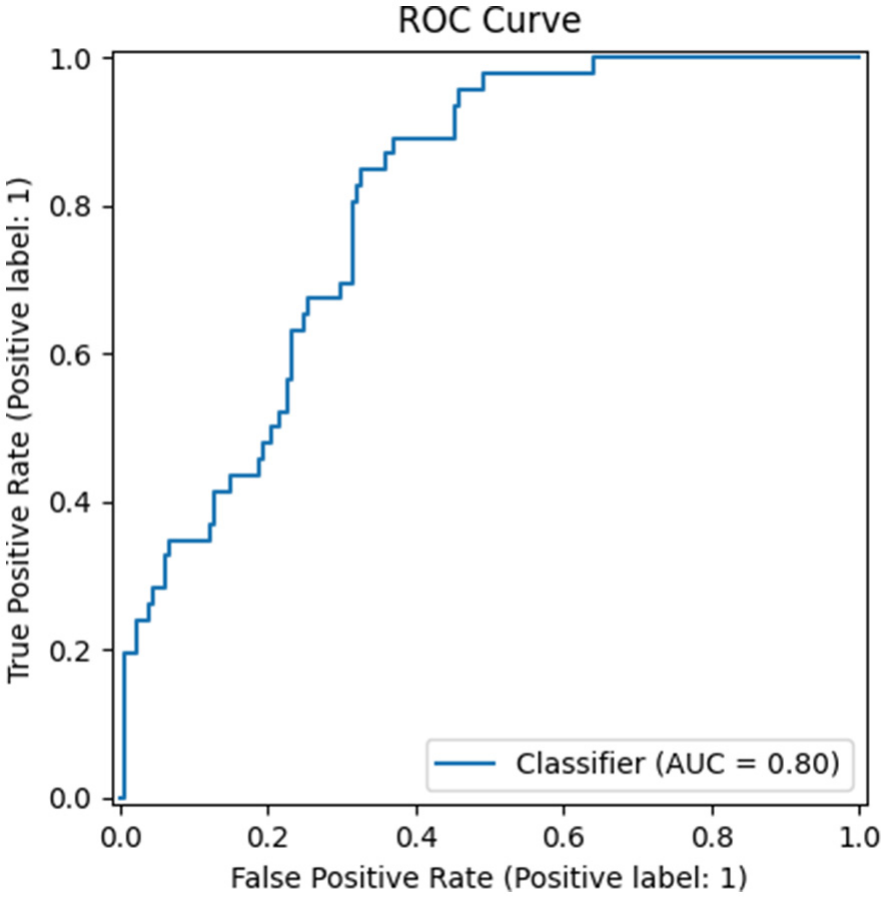
ROC curve of the random forest model on the external validation set.

In addition, the external validation of the random forest model showed that the AUC value was 0.80, and the ROC curve was shown in Figure 4, indicating that the internal validation effect was better than the external validation effect.

## 4 Discussion

This study showed that the random forest model had the best performance in the prediction of metabolic syndrome in nurses (AUC = 0.879 in test set), which was significantly better than traditional models such as Logistic regression (AUC = 0.672). This strength stems from its effective capture of non-linear associations of key factors, as shown by the feature importance map in Figure 3, where BMI and job burnout were identified as core influencing factors. For example, random forests had the highest SHAP values for BMI, and Logistic regression did not adequately address such complex interaction effects. Therefore, the ensemble learning algorithm can more accurately reflect the multi-factor pathogenic mechanism of metabolic syndrome, provide a reliable tool for high-risk nurse screening, and support the implementation of targeted health intervention.

Multivariate Logistic regression analysis showed that nurses' educational background, number of children, frequency of tea/coffee intake, family history of cardiovascular disease and other variables were not statistically associated with the risk of metabolic syndrome (*P* > 0.05). The lack of correlation may be due to the characteristics of sample distribution: only 0.56% of nurses had master's degree or above, 1.11% of nurses were divorced or widowed, and the night shift load of specific departments varied significantly. Such concentration phenomenon is in line with the characteristics of nurses in tertiary hospitals, who are mainly young women and with bachelor's degree. Their occupational stress and sleep disorder may cover up the independent effects of some variables.

This study confirmed BMI as the most influential core predictor of metabolic syndrome in nurses. Elevated BMI significantly increases the risk of insulin resistance and dyslipidemia, which is consistent with the research conclusions of Wang et al. (18) on central obesity and metabolic disorders in nurses. Previous studies have compared the importance differences between traditional clinical indicators and machine learning model features, and the results show that the predictive power of BMI in the random forest model is significantly better than that of working years in the Logistic regression model, especially for the group of shift nurses. The two studies consistently confirmed the central role of BMI in predicting the metabolic syndrome, although they used traditional statistical methods and machine-learning techniques, respectively. Therefore, BMI monitoring should be the primary indicator of nurses' health management. Its simple measurement operation, objective results and strong biological correlation with metabolic indicators provide an efficient tool for large-scale screening of high-risk groups.

Job burnout in this study was established as the second largest predictor of metabolic syndrome in nurses. This result is consistent with the conclusions of most studies, suggesting that burnout may promote metabolic abnormalities through chronic stress-mediated hormonal disturbances such as elevated cortisol or a proinflammatory state (7, 19, 20). de Souza et al. (21) found that long-term occupational stress is significantly associated with insulin resistance and abdominal obesity, while persistent burnout may weaken the individual's ability to manage health behaviors (such as reduced exercise or uncontrolled eating), thus aggravating metabolic risk. Chico-Barba et al. (22) found through an observational study that job burnout was significantly and positively correlated with the risk of metabolic syndrome, and the higher the degree of job burnout, the higher the prevalence of metabolic abnormal indicators (such as abdominal obesity and hypertension).

Compared with the traditional Logistic regression model, the machine learning model significantly improved the prediction performance of metabolic syndrome. Logistic regression is difficult to capture the complex interaction of risk factors due to its limited explanatory power and only linear association identification. Therefore, this study compares multiple machine learning algorithms aiming to mine key feature interaction effects through non-linear modeling. The random forest model achieved the best performance (AUC of test set = 0.879, accuracy rate 0.869), and its core predictors included BMI, job burnout, shift system, age and other 9 characteristics. Tan et al. (23) also pointed out that in terms of predicting diabetic microvascular and macrovascular complications, the random forest model outperformed traditional non-machine learning methods in many studies. Zhang et al. (24) used a machine learning model to analyze the association between occupational stress and metabolic syndrome, and verified the high-precision predictive ability of psychological factors in the early warning of metabolic risk. These studies collectively show that machine learning shows significant advantages over traditional methods in the field of health risk prediction. Although this study confirms the advantages of machine learning in health prediction, it is still necessary to expand the sample size in the future to optimize the generalization ability of the model.

## 5 Implications for nursing and health policy

The strong association between job burnout and metabolic syndrome highlights the critical link between psychological wellbeing and physical health. Healthcare institutions must recognize burnout prevention not merely as a wellness initiative but as a core metabolic disease prevention strategy. Policymakers should consider nurse-to-patient ratio regulations that account for both patient safety outcomes and nurse metabolic health impacts. The model's feature importance underscores the need for systemic changes including adequate staffing, psychological support services, and workplace wellness programs with measurable metabolic outcomes.

## 6 Relevance to clinical practice

The results of this study show that the risk of metabolic syndrome in nurses is mainly affected by a variety of factors, and these factors show dynamic changes in the career of nurses. In order to fully grasp the changing trend of the risk of metabolic syndrome in nurses, it is necessary to deeply analyze its potential influencing factors and pay attention to the evolution of these factors over time. In addition, by quantitatively analyzing the risk factors of metabolic syndrome in nurses, nursing managers can obtain more accurate and reliable risk assessment. Finally, nursing managers can use machine learning techniques (such as random forest models) to enhance their ability to predict future risks, continuously integrate new data and optimize models, so as to effectively improve the work efficiency and resource allocation of clinical nursing teams to adapt to changing nursing needs and medical environments.

## 7 Conclusion

The risk of metabolic syndrome in nurses is affected by many factors, including demographic characteristics, lifestyle and occupational psychological factors. Although previous studies have explored the influencing factors of metabolic syndrome in nurses, this study further clarified the key factors affecting metabolic syndrome in nurses through systematic review of relevant literature at home and abroad combined with cross-sectional survey research, and constructed a risk prediction model based on a variety of machine learning algorithms, which provided a new perspective for existing research. The results of this study help nursing managers to accurately identify high-risk groups of nurses, so as to formulate more targeted health management strategies. In addition, given the dynamic nature of the model, it is recommended to continuously optimize the prediction model according to the occupational exposure characteristics of nurses, the changes of working environment, and the evolution of individual health status, so as to improve its applicability and accuracy in different nursing scenarios.

## 8 Limitations

The data set of this study may only contain limited variables and characteristics, which does not comprehensively cover all factors that may affect the risk of metabolic syndrome in nurses. In future studies, the inclusion of more comprehensive data should be considered to improve the predictive power and explanatory power of the model. In addition, stress biomarkers such as salivary cortisol were not assessed, and the cross-sectional design cannot rule out reverse causality. In the future, longitudinal cohorts and physiological indicators need to be combined to verify the causal pathway and intervention potential.

## Data Availability

The raw data supporting the conclusions of this article will be made available by the authors, without undue reservation.
